# Non-Invasive Brain Stimulation and Artificial Intelligence in Communication Neuroprosthetics: A Bidirectional Approach for Speech and Hearing Impairments

**DOI:** 10.3390/brainsci15050449

**Published:** 2025-04-25

**Authors:** Thorsten Rudroff

**Affiliations:** Turku PET Centre, University of Turku, Turku University Hospital, 20520 Turku, Finland; thrudr@utu.fi

**Keywords:** artificial intelligence, neuroprosthetics, speech, hearing, brain stimulation

## Abstract

This perspective paper introduces a novel bidirectional framework for communication neuroprosthetics that integrates non-invasive brain stimulation (NIBS) with artificial intelligence (AI) to address both speech production and auditory processing impairments. Traditional approaches typically target only one aspect of communication, while this framework supports the complete communication cycle. The integration of transcranial direct current stimulation (tDCS) and transcranial alternating current stimulation (tACS) with advanced AI algorithms enables the personalized, adaptive enhancement of neural signals for both speaking and listening functions. This paper examines current evidence for NIBS efficacy in communication disorders, explores AI innovations in neural signal processing, and discusses implementation considerations for closed-loop systems. This bidirectional approach has the potential to provide more natural, effective communication support while promoting neuroplasticity for long-term recovery. The framework presented offers a roadmap for developing accessible communication interventions that could significantly improve the quality of life for individuals with speech and hearing impairments resulting from neurological conditions.

## 1. Introduction

Effective communication is fundamental to human connection, social participation, and quality of life. For millions of individuals with speech and hearing impairments resulting from neurological conditions, trauma, or congenital disorders, the inability to communicate naturally can lead to profound isolation, diminished autonomy, and psychological distress [1,2]. Recent advances in neurotechnology and artificial intelligence have created unprecedented opportunities to restore communicative functions through direct brain–computer interfaces, yet significant challenges remain in developing systems that are both effective and accessible [3,4].

In essence, bidirectional communication refers to supporting both aspects of human conversation: speaking and listening. Natural communication involves a continuous cycle of expressing our thoughts (speech production) and understanding others (auditory processing). Traditional approaches to communication disorders often address only one of these directions, either helping someone speak or helping them hear. The bidirectional framework proposed in this perspective integrates both directions into a unified system. Using AI-guided brain stimulation, the system can enhance neural activity related to speech production when the user is trying to speak and then seamlessly switch to enhancing auditory processing when the user is listening. This adaptive approach provides comprehensive support across the entire communication cycle, potentially enabling more natural and effective interactions than unidirectional approaches alone.

The landscape of communication neuroprosthetics has evolved dramatically over the past decade. While early brain–computer interfaces (BCIs) for communication relied on slow, discrete selection methods that yielded only a few words per minute [5,6], recent breakthroughs have demonstrated the feasibility of decoding continuous speech directly from neural activity. Most notably, Littlejohn et al. [7] reported a groundbreaking advance in Nature Neuroscience with their streaming brain-to-voice neuroprosthesis. Their system used high-density surface recordings from the speech sensorimotor cortex of a participant with severe paralysis and anarthria to drive a continuously streaming speech synthesizer. Through deep learning recurrent neural network transducer models, they achieved online large-vocabulary speech synthesis personalized to the participant’s pre-injury voice, with neural decoding in 80 ms increments. This system enabled naturalistic communication with minimal delay, addressing the social isolation often experienced by individuals with paralysis. Importantly, their framework also generalized to other interfaces including single-unit recordings and electromyography, suggesting broader applicability beyond their specific implementation. In parallel, recent work by Rudroff et al. [8] has demonstrated the promising potential of AI-guided transcranial direct current stimulation (tDCS) for Long COVID management, providing a valuable precedent for considering the integration of AI with non-invasive brain stimulation for neurological disorders. Their framework for personalized neuromodulation through AI offers insights that may be particularly relevant to addressing the heterogeneous manifestations of communication disorders [9,10].

Concurrently, significant advances have been made in non-invasive brain stimulation (NIBS) techniques such as transcranial direct current stimulation (tDCS), transcranial alternating current stimulation (tACS), and transcranial magnetic stimulation (TMS) [11]. These approaches have demonstrated promising effects on neuroplasticity, cortical excitability, and functional rehabilitation across various neurological domains [12,13]. In particular, multiple studies have shown that targeted NIBS can enhance both speech motor learning [14,15] and auditory processing capabilities [16,17,18,19].

The parallel explosion of artificial intelligence, particularly in deep learning approaches for neural signal processing, has transformed the computational foundations of neuroprosthetic systems. Modern machine learning algorithms can extract meaningful patterns from complex, noisy neural signals with unprecedented accuracy [20,21], learn to adapt to individual neural signatures [22], and generate naturalistic outputs whether in the form of synthesized speech [23] or optimized auditory stimuli [24].

This perspective proposes that the integration of non-invasive brain stimulation with AI-driven communication neuroprosthetics presents a promising frontier for bidirectional communication restoration. This convergence could potentially address both speech production and auditory processing impairments through complementary mechanisms: NIBS to enhance neuroplasticity and optimize neural signal characteristics and AI to decode or encode complex neural patterns with personalized precision.

This perspective explores the theoretical foundations, current evidence, technical considerations, and future directions for this integrated approach. The examination covers both the speech production and auditory processing domains, with an emphasis on how non-invasive methodologies might eventually provide accessible alternatives or complements to invasive neuroprosthetic systems. By focusing on non-invasive approaches, this paper aims to expand the potential reach of these technologies to broader clinical populations and settings where invasive interventions may not be feasible, desired, or necessary [25,26].

The bidirectional framework proposed acknowledges that effective communication requires both expressive and receptive capabilities and that advances in one domain may inform and enhance approaches in the other. Through this integrative lens, this perspective envisions communication neuroprosthetics that holistically address the complete cycle of human interaction, potentially transforming the lives of individuals with speech and hearing impairments.

## 2. Current State of Communication Neuroprosthetics

### 2.1. Invasive Approaches: Context and Contributions

While this perspective focuses on non-invasive approaches, understanding the contributions of invasive methods provides important context. Invasive BCI approaches for communication involve recording neural activity via electrodes implanted on the surface of the brain (electrocorticography, ECoG) or directly within brain tissue (intracortical microelectrodes).

Hochberg et al. [10] demonstrated high-performance neural control of robotic devices using intracortical recordings, highlighting the precision possible with direct neural recordings. In the communication domain, Brumberg et al. [27] showed that intracortical recordings from speech motor areas could be used to decode attempted speech movements in real time.

The most significant advancements in invasive speech BCIs came with the work of Anumanchipalli et al. [24], who demonstrated speech synthesis from neural activity recorded from the sensorimotor cortex during attempted speech. Building on this foundation, Littlejohn et al. [7] achieved continuous streaming speech synthesis with naturalistic quality personalized to the user’s pre-injury voice. This represents the current state of the art in invasive communication neuroprosthetics.

While these invasive approaches demonstrate remarkable capabilities, they require neurosurgical intervention with associated risks and limitations in longevity and stability, restricting their widespread applicability [9]. However, the neural decoding principles developed in these studies provide valuable insights for advancing non-invasive approaches.

### 2.2. Non-Invasive Approaches for Speech and Hearing Applications

Non-invasive communication neuroprosthetics primarily utilize electroencephalography (EEG), functional near-infrared spectroscopy (fNIRS), and magnetoencephalography (MEG) to capture neural signals without requiring surgical implantation.

#### 2.2.1. EEG-Based Approaches for Speech Production

Current EEG-based communication systems employ several paradigms:

**Motor Imagery and P300**: Systems leverage imagined movements to modulate sensorimotor rhythms or detect event-related potentials, enabling discrete selections for communication [28,29]. While functional, these approaches remain limited to selection-based communication rather than natural speech production.
**Continuous Speech Decoding**: Recent work has pursued the continuous decoding of speech features from EEG. Nguyen et al. [30] demonstrated the decoding of phonemic information from EEG during covert speech, while Papadimitriou et al. [31] utilized transformer-based deep learning models to reconstruct continuous speech features from EEG. Though promising, these approaches currently achieve only limited vocabulary reconstruction with moderate intelligibility.

#### 2.2.2. fNIRS and Multimodal Approaches

Functional near-infrared spectroscopy measures hemodynamic responses associated with neural activity, offering complementary information to electrophysiological methods. Recent work by Hong et al. [32] demonstrated that hybrid EEG-fNIRS systems can improve classification accuracy for communication-related mental tasks compared to either modality alone.

#### 2.2.3. Non-Invasive Approaches for Hearing Applications

Non-invasive technologies for hearing typically focus on enhancing auditory processing rather than directly replacing it:

**Auditory Attention Decoding**: O’Sullivan et al. [33] demonstrated the feasibility of decoding auditory attention from EEG in multi-speaker environments, forming the basis for cognitive hearing aids that enhance attended speech. Recent advances by Alickovic et al. [34] have improved real-time decoding accuracy and reduced the latency of auditory attention detection.
**Auditory Processing Enhancement**: Transcranial stimulation shows promise for modulating auditory processing. Heimrath et al. [17] demonstrated that tACS at specific frequencies can enhance temporal auditory processing, while Vanneste et al. [18] reported the differential effects of various transcranial stimulation approaches on tinnitus perception.

### 2.3. Current Limitations and Recent Breakthroughs

Despite advances, non-invasive communication neuroprosthetics face several key limitations:

**Signal Quality Challenges**: EEG signals related to speech processes are often obscured by noise, with signal-to-noise ratios typically below 1:10 [35], making it difficult to extract clean neural signals related to speech production and perception. Limited spatial resolution (2–3 cm) restricts the ability to distinguish activity from adjacent cortical regions involved in speech production and perception [36].
**Performance Limitations**: Current non-invasive approaches achieve information transfer rates of only 0.5–3 words per minute in practical scenarios, far below natural speech rates of 120–180 words per minute. Vocabulary limitations also persist, with state-of-the-art EEG-based systems decoding only 50–100 distinct words with moderate accuracy [37].
**Individual Variability**: Substantial inter-individual and session-to-session variability necessitates frequent recalibration. Transfer learning across subjects for speech imagery tasks achieves only 40–60% of the performance of subject-specific models [38], highlighting the need for personalization.

Recent breakthroughs, however, suggest promising paths forward:

**Advanced Machine Learning**: Krishna et al. [39] demonstrated the continuous reconstruction of intelligible speech from EEG using convolutional-recurrent neural networks. Willett et al. [40] introduced self-supervised pre-training approaches that significantly reduce the amount of individual training data required for effective speech decoding.
**Integration of Recording and Stimulation**: Conde et al. [41] demonstrated a closed-loop system integrating EEG-based attention detection with targeted auditory enhancement using transcranial alternating current stimulation. This system detected the attended speaker in multi-speaker environments and selectively enhanced the processing of that speech stream.
**Multimodal Integration**: Putze et al. [42] showed that combining EEG, fNIRS, and eye-tracking improved the classification accuracy of speech imagery by 23% compared to EEG alone. Chen et al. [43] demonstrated that incorporating subtle facial muscle activity with EEG significantly improved speech decoding accuracy in motor-impaired individuals.

These advances, while promising, have largely developed along separate tracks for speech production and auditory processing. The bidirectional integration of these approaches, supported by AI-guided non-invasive brain stimulation, represents an underexplored frontier with significant therapeutic potential. The following sections will explore how such integration might be realized and the potential benefits it could offer for individuals with communication disorders.

## 3. Non-Invasive Brain Stimulation: Mechanisms and Applications

Non-invasive brain stimulation (NIBS) encompasses techniques that modulate neural activity without requiring surgical intervention. This section briefly reviews the key NIBS modalities relevant to communication neuroprosthetics and their applications in speech and auditory processing.

### 3.1. NIBS Modalities

**Transcranial Direct Current Stimulation (tDCS)** applies weak constant electrical currents (typically 1–2 mA) via scalp electrodes to modulate cortical excitability. Anodal stimulation generally increases neuronal excitability, while cathodal stimulation typically decreases it [11,12]. The relatively low cost, portability, and ease of administration of tDCS make it particularly suitable for widespread clinical applications in communication disorders.
**Transcranial Alternating Current Stimulation (tACS)** delivers oscillating electrical currents at specific frequencies, with the potential to entrain brain oscillations to the stimulation frequency. This approach shows particular promise for modulating oscillatory activity relevant to speech and auditory processing [13], which often depends on precise temporal dynamics across multiple frequency bands.

### 3.2. Applications in Speech Production and Auditory Processing

#### 3.2.1. Speech Production

Multiple studies have demonstrated tDCS efficacy for enhancing speech production. Marangolo et al. [15] showed that anodal tDCS over the left inferior frontal cortex significantly improved speech production in chronic aphasia patients. These improvements in articulatory accuracy and naming abilities persisted for at least four weeks post-stimulation.

For speech fluency, Chesters et al. [16] found that anodal stimulation over left inferior frontal regions combined with fluency training reduced dysfluency in people who stutter, with effects persisting beyond the stimulation period. These findings suggest that appropriately targeted stimulation can enhance both speech motor learning and execution.

#### 3.2.2. Auditory Processing

In the auditory domain, Heimrath et al. [17] demonstrated that tACS synchronized to individual alpha frequencies can enhance temporal auditory processing, potentially improving speech discrimination in challenging environments. This approach shows particular promise for addressing difficulties in auditory processing that may contribute to communication disorders.

Vanneste et al. [18] found that different transcranial stimulation approaches have varying efficacy for tinnitus, depending on stimulation parameters and specific tinnitus characteristics. These findings highlight the potential for personalized NIBS approaches based on individual symptom profiles.

### 3.3. Comparative Efficacy and Individual Variability

The efficacy of NIBS varies considerably based on the targeted neural process and individual factors. While tDCS offers broader neuromodulation suitable for enhancing speech motor learning, tACS potentially offers greater specificity for auditory processing by targeting specific oscillatory frequencies [13].

Significant inter-individual variability in response to NIBS has been documented, with factors including anatomy, genetics, and neurophysiological state influencing outcomes [12]. This variability underscores the potential benefit of AI-guided personalization, as a one-size-fits-all approach is unlikely to be optimal across diverse individuals with communication disorders.

Current evidence supports NIBS as a promising approach for modulating neural activity in both speech production and auditory processing domains. However, the heterogeneity of communication disorders and individual variation in response to stimulation highlight the need for more personalized, adaptive approaches—precisely the challenge that AI-guided NIBS aims to address.

## 4. Artificial Intelligence in Neural Decoding

Artificial intelligence, particularly machine learning and deep learning approaches, has transformed neural signal processing for communication applications. This section highlights key AI advances relevant to bidirectional communication neuroprosthetics.

### 4.1. Evolution of AI in BCI Applications

The application of AI to brain–computer interfaces has progressed from simple classification algorithms to sophisticated deep learning approaches. Early systems relied primarily on linear discriminant analysis and support vector machines, which offered computational efficiency but struggled with the high-dimensional, non-stationary nature of neural signals [21].

Recent advances in deep learning have addressed many limitations of classical approaches. Convolutional neural networks (CNNs) have proven particularly effective for spatial pattern recognition in neural data, while recurrent neural networks (RNNs) excel at capturing the temporal dynamics critical for speech decoding [22]. Transformer-based architectures, originally developed for natural language processing, have recently shown promise for neural decoding due to their ability to capture long-range dependencies in time-series data [31].

### 4.2. Key AI Innovations for Neural Signal Processing

Several key AI innovations have significantly advanced neural signal processing:

**End-to-end learning** eliminates manual feature engineering by learning optimal representations directly from raw neural signals. This approach has proven particularly valuable for decoding complex communication signals where relevant features may not be obvious [21].
**Transfer learning** enables knowledge transfer across subjects, sessions, and tasks, potentially reducing calibration requirements. Willett et al. [40] demonstrated that self-supervised pre-training on large speech corpora can significantly reduce the subject-specific neural data needed for effective speech decoding.
**Multimodal integration** techniques allow AI models to fuse information from complementary data sources. Chen et al. [43] showed that incorporating minimal sEMG signals with EEG significantly improved speech decoding accuracy in motor-impaired individuals.

### 4.3. AI for Personalization and Adaptation

AI techniques have demonstrated significant potential for the personalization and adaptation of neural decoding systems:

**Subject-specific optimization** approaches use techniques such as Bayesian optimization to efficiently search for the high-dimensional parameter space of decoding models for individual users, addressing the substantial inter-individual variability observed in neural responses [22].
**Online adaptation** algorithms continuously update model parameters during use, addressing the non-stationarity of neural signals across time. Such approaches are crucial for maintaining performance across sessions without requiring frequent recalibration [21].
**Reinforcement learning** frameworks enable the optimization of decoding strategies based on implicit or explicit user feedback, potentially allowing systems to improve naturally through use [23].

### 4.4. AI-Guided Brain Stimulation

Beyond neural decoding, AI approaches have begun to demonstrate value for optimizing brain stimulation parameters. Rudroff et al. [8] highlighted the potential of AI-guided transcranial direct current stimulation for personalized neuromodulation in neurological conditions.

AI-guided stimulation can address several key challenges:

**Individual variability**: Machine learning models can predict optimal stimulation parameters based on individual brain anatomy, functional organization, and response characteristics.
**Parameter optimization**: Reinforcement learning approaches can efficiently explore high-dimensional stimulation parameter spaces (intensity, duration, montage, timing) to identify optimal protocols.
**Adaptive stimulation**: Closed-loop systems can dynamically adjust stimulation parameters based on ongoing neural activity and behavioral performance.

The integration of AI-guided neural signal processing with AI-optimized brain stimulation offers a powerful framework for addressing the bidirectional nature of communication disorders. By combining these approaches, systems can potentially provide personalized, adaptive support for both speech production and auditory processing, addressing the complete communication cycle in a coordinated manner.

## 5. Bidirectional Applications of NIBS and AI in Communication Disorders

In essence, bidirectional communication refers to supporting both aspects of human conversation: speaking and listening. Natural communication involves a continuous cycle of expressing our thoughts (speech production) and understanding others (auditory processing). Traditional approaches to communication disorders often address only one of these directions—either helping someone speak or helping them hear. The bidirectional framework proposed in this perspective integrates both directions into a unified system. Using AI-guided brain stimulation, the system can enhance neural activity related to speech production when the user is trying to speak and then seamlessly switch to enhancing auditory processing when the user is listening. This adaptive approach provides comprehensive support across the entire communication cycle, potentially enabling more natural and effective interactions than unidirectional approaches alone.

### 5.1. Speech Production Domain

#### 5.1.1. Enhanced Neural Signal Acquisition Through Targeted Neuromodulation

Non-invasive brain stimulation can significantly enhance the quality of neural signals acquired for speech decoding by modulating cortical excitability in speech-relevant regions. tDCS applied over the left inferior frontal and motor cortices has been shown to increase the signal-to-noise ratio in neural recordings, potentially improving decoding accuracy.

Fertonani and Miniussi [12] demonstrated that anodal tDCS over speech motor areas temporarily increases cortical excitability, resulting in more robust motor-related neural signals. This enhancement can be particularly valuable for individuals with weak or degraded neural signals due to neurological conditions. For example, in post-stroke aphasia, where neural activity in language areas is often diminished, the strategic application of tDCS has been shown to increase activation in perilesional tissue, potentially enhancing signal detectability for BCIs [15].

AI algorithms can optimize this neuromodulation-enhanced signal acquisition in several ways:

**Spatial Targeting Optimization**: Deep learning models can analyze individual neuroimaging data to identify optimal stimulation targets based on functional and structural connectivity patterns. Khadka et al. [44] demonstrated that neural networks can predict individualized electric field distributions in tDCS, potentially enabling more precise targeting of speech-related neural circuits. This approach addresses the significant inter-individual variability in brain anatomy and functional organization that impacts stimulation efficacy.
**Temporal Protocol Optimization**: Reinforcement learning algorithms can optimize stimulation timing relative to speech decoding tasks. Moses et al. [45] showed that the timing of stimulation relative to task performance significantly impacts efficacy. By systematically exploring different timing protocols and learning from outcomes, AI can identify optimal temporal patterns for enhancing neural signal acquisition in individual users.
**Parameter Personalization**: Bayesian optimization approaches can efficiently navigate the high-dimensional parameter space of stimulation (intensity, duration, electrode configuration) to identify optimal settings for each user. Lorenz et al. [46] demonstrated the efficacy of this approach for optimizing transcranial electrical stimulation parameters in cognitive enhancement, and similar methods could be applied to optimize parameters for speech signal enhancement.

**Figure 1:**
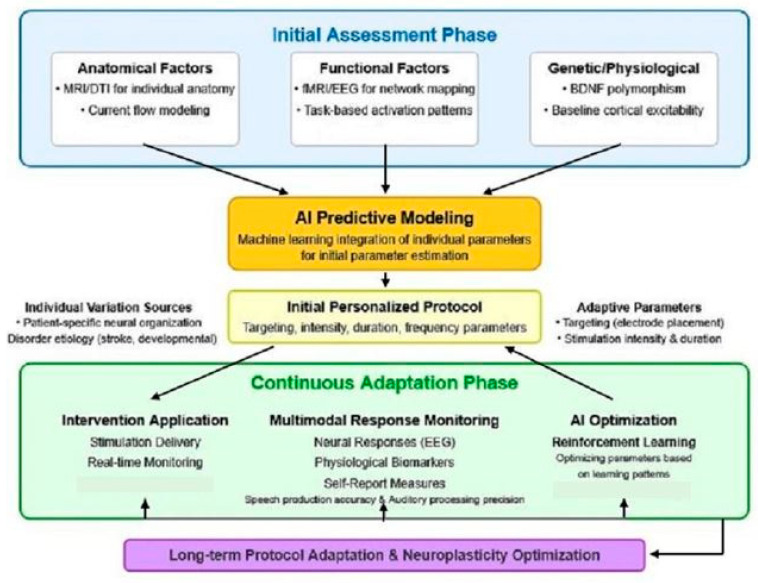
AI-driven personalization framework for neuromodulation.

Figure 1 illustrates the AI-driven personalization framework for optimizing neuromodulation parameters in communication neuroprosthetics. This bidirectional approach integrates multiple streams of individual patient data to develop and continuously refine stimulation protocols. The framework operates in two key phases: an initial assessment phase that establishes baseline personalized parameters based on anatomical [47], functional [32], and genetic/physiological [48] factors and a continuous adaptation phase that implements reinforcement learning to optimize parameters based on ongoing neural, behavioral, and self-reported responses. This closed-loop system addresses the significant inter-individual variability in stimulation response that has limited the efficacy of standardized protocols, potentially enabling more precise and effective neuromodulation for both speech production and auditory processing enhancement.

The upper blue section represents the Initial Assessment Phase, where individual factors are analyzed to generate starting parameters. The lower green section depicts the Continuous Adaptation Phase, which implements real-time monitoring [41] and reinforcement learning [46] to optimize stimulation parameters based on communication task performance. Arrows indicate data flow and feedback loops that enable the progressive refinement of the stimulation protocol. The referenced studies provide empirical support for specific components of the framework.

#### 5.1.2. Neuroplasticity Facilitation for Improved Motor Learning

Beyond immediate signal enhancement, NIBS can facilitate neuroplasticity to improve speech motor learning over time. When applied during or immediately before speech training, tDCS has been shown to enhance the acquisition and retention of speech motor skills.

Marangolo et al. [15] demonstrated that anodal tDCS over the left inferior frontal cortex significantly improved articulation learning in chronic aphasia patients. Similarly, Chesters et al. [16] found that combining tDCS with fluency training produced greater improvements in people who stutter than training alone. These findings suggest that appropriately timed and targeted stimulation can enhance the neuroplastic changes underlying speech motor learning.

AI approaches can optimize this neuroplasticity facilitation through:

**Adaptive Learning Rate Modulation**: Machine learning algorithms can track individual learning curves and adjust stimulation parameters to optimize the rate of skill acquisition. By modeling the relationship between stimulation parameters and learning outcomes, AI can identify the optimal stimulation protocol for each stage of the learning process.
**Personalized Difficulty Progression**: AI can dynamically adjust task difficulty based on performance and neurophysiological markers of learning, ensuring optimal challenge levels. This approach leverages principles of optimal learning theory, where maintaining an appropriate challenge level maximizes learning efficiency. When combined with optimized NIBS, this could significantly accelerate speech motor learning.
**Multimodal Biomarker Integration**: Deep learning models can integrate multiple biomarkers of neuroplasticity (e.g., changes in EEG connectivity, behavioral performance improvements) to guide stimulation protocols. Gharabaghi et al. [49] demonstrated that combining neural and behavioral markers provides more sensitive detection of stimulation effects than either alone, potentially enabling more precise tuning of NIBS parameters for optimal neuroplasticity induction.

#### 5.1.3. Closed-Loop Systems for Speech Decoding

Perhaps the most promising application of AI-guided NIBS for speech production is the development of closed-loop systems that dynamically adjust stimulation based on ongoing neural activity and task performance. These systems continuously monitor relevant biomarkers and modulate stimulation parameters in real time to optimize both immediate performance and long-term learning.

Conde et al. [41] demonstrated a closed-loop system for auditory processing that dynamically adjusted tACS parameters based on EEG markers of attention. A similar approach could be implemented for speech production, where stimulation parameters are adjusted based on markers of speech motor planning and execution.

AI enables sophisticated closed-loop control through:

**Predictive Modeling**: Deep learning models can predict upcoming difficulties in speech production based on neural precursors, allowing the preemptive adjustment of stimulation to facilitate challenging speech segments. For example, by detecting the neural signatures that precede articulatory difficulties, the system could increase stimulation intensity or shift targeting to provide timely facilitation.
**Multi-objective Optimization**: Reinforcement learning algorithms can balance multiple objectives such as immediate performance improvement, long-term learning, and comfort. This approach recognizes the complex tradeoffs in neuromodulation, where parameters that maximize immediate performance might differ from those that optimize long-term skill acquisition.
**Hybrid System Integration**: Machine learning approaches can integrate NIBS with other intervention modalities (e.g., auditory feedback, visual cues) to create comprehensive closed-loop systems. By considering multiple intervention channels simultaneously, AI can identify synergistic combinations that exceed the efficacy of any single approach.

**Figure 2:**
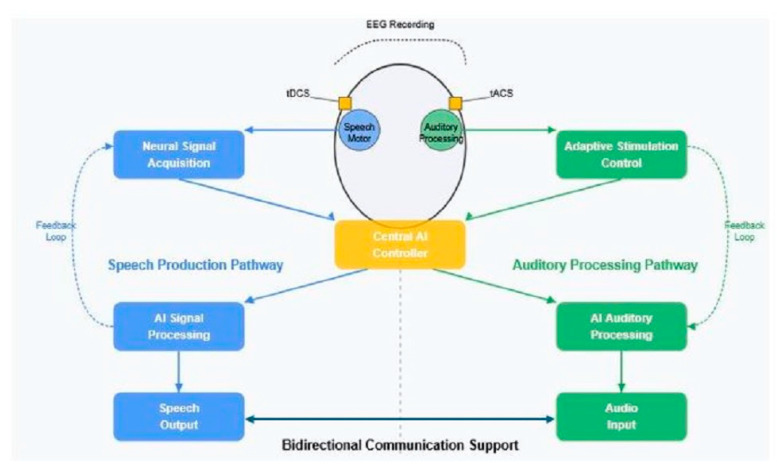
Conceptual framework for bidirectional communication neuroprosthetics integrating non-invasive neural recording, AI-driven signal processing, and adaptive NIBS for both speech production and auditory processing enhancement.

A conceptual model for such a closed-loop system is presented in Figure 2, illustrating the integration of neural signal acquisition, AI-driven signal processing, and adaptive NIBS control for speech production enhancement.

### 5.2. Auditory Processing Domain

#### 5.2.1. Enhancing Auditory Cortex Receptivity Through NIBS

Non-invasive brain stimulation offers significant potential for enhancing auditory processing by modulating the excitability and oscillatory dynamics of auditory cortical regions. This enhancement can benefit individuals with central auditory processing disorders, age-related hearing decline, and hearing impairments that are not fully addressed by conventional amplification.

Heimrath et al. [17] demonstrated that tACS can modulate temporal processing in the auditory system by entraining neural oscillations to behaviorally relevant frequencies. This approach shows particular promise for enhancing speech discrimination in noisy environments, where temporal processing is critical for segregating target speech from background noise.

AI can optimize auditory enhancement through:

**Frequency-Specific Targeting**: Deep learning models can identify individualized oscillatory signatures associated with optimal auditory processing, allowing targeted training using tACS. Gherman et al. [50] showed that individual alpha frequency tACS over temporal regions enhances phoneme discrimination, but optimal frequencies vary across individuals. AI can efficiently identify these individual optima through systematic exploration and learning.
**Attention-Based Modulation**: Machine learning algorithms can detect neural markers of auditory attention and dynamically adjust stimulation to enhance the processing of attended auditory streams. O’Sullivan et al. [33] demonstrated that auditory attention can be decoded from EEG, providing a potential control signal for adaptive NIBS. By selectively enhancing the neural processing of attended speech, this approach could significantly improve listening performance in complex acoustic environments.
**Cross-Modal Integration Enhancement**: Deep neural networks can identify patterns of audiovisual integration and optimize stimulation to enhance multisensory processing. Many individuals with hearing impairments rely on visual cues (e.g., lip-reading) to supplement auditory information. NIBS protocols that enhance cross-modal integration, guided by AI models that understand individual multisensory processing patterns, could significantly improve overall communication ability.

#### 5.2.2. AI-Driven Optimization of Auditory Stimulus Encoding

Beyond direct neural modulation, AI can optimize the transformation of acoustic signals into patterns of neural stimulation that maximize intelligibility and reduce listening effort.

Han et al. [51] developed a system that continuously monitors auditory event-related potentials to optimize acoustic feature processing for individual users. Their approach dynamically adjusted frequency amplification and noise suppression based on neural markers of processing difficulty, demonstrating significant improvements in speech comprehension.

AI approaches enable the sophisticated optimization of auditory stimulus encoding through:

**Personalized Feature Mapping**: Neural networks can learn mappings between acoustic features and optimal stimulation parameters for individual users. This approach recognizes the significant individual differences in auditory processing and allows for highly personalized enhancement strategies.
**Context-Adaptive Processing**: Reinforcement learning algorithms can identify optimal stimulus processing strategies for different acoustic environments and listening goals. For example, parameters that optimize speech understanding in quiet environments might differ from those ideal for noisy settings or music appreciation. By learning these context-specific optimizations, AI can provide adaptive enhancement that seamlessly adjusts to changing environments.
**Cognitive Load Minimization**: Machine learning models can monitor markers of cognitive effort and adjust processing to minimize listening effort while maintaining intelligibility. This approach recognizes that speech understanding involves both bottom-up signal processing and top-down cognitive resources. By optimizing for cognitive efficiency, these systems can reduce the fatigue often associated with effortful listening.

#### 5.2.3. Closed-Loop Auditory Enhancement Systems

The integration of neural recording, AI processing, and NIBS creates the potential for closed-loop systems that continuously optimize auditory processing based on neural and behavioral feedback.

Conde et al. [41] demonstrated a closed-loop system that integrated EEG-based attention detection with tACS to enhance auditory processing. Their system detected the attended speaker in a multi-speaker environment and selectively enhanced the processing of that speech stream through phase-synchronized stimulation.

**Figure 3:**
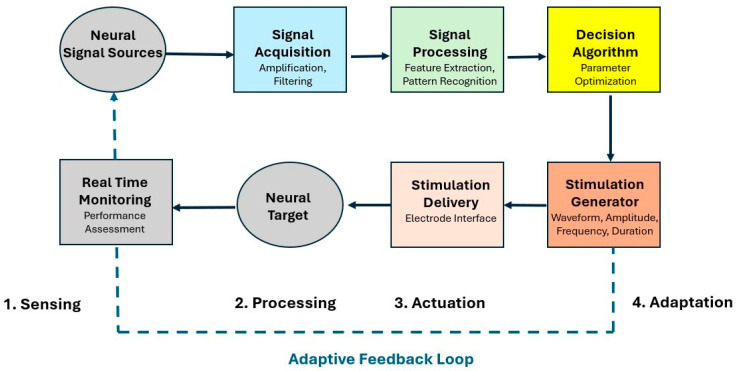
Closed-loop neural signal processing system.

Figure 3 presents a comprehensive framework for a bidirectional closed-loop neural processing system that addresses both speech production and auditory processing. For hearing enhancement specifically, the system continuously monitors the neural activity associated with auditory attention and comprehension. As illustrated in the diagram, neural signals from auditory cortical regions are acquired and processed through AI-driven signal analysis, which can identify markers of processing difficulty or attention focus. Based on these signals, the system dynamically adjusts stimulation parameters delivered to auditory processing regions, potentially enhancing frequency-specific neural activity or facilitating attention to relevant speech streams in complex listening environments. The adaptive nature of this closed-loop approach allows the system to provide personalized auditory enhancement that responds to changing acoustic environments and listening demands, potentially reducing listening effort while maintaining intelligibility. This represents a significant advance over static hearing enhancement approaches that cannot adjust to the dynamic nature of real-world listening situations.

AI enables sophisticated closed-loop auditory enhancement through:

**Adaptive Stimulation Timing**: Deep learning models can predict optimal phases of ongoing neural oscillations for stimulus presentation or stimulation delivery. For example, by identifying the phase of theta oscillations most receptive to speech input, the system could time stimulation to maximize the impact on speech processing.
**Dynamic Difficulty Adjustment**: Reinforcement learning algorithms can continuously adjust the level of signal enhancement based on performance and neural markers of processing difficulty. This approach ensures that users remain appropriately challenged, maximizing the potential for perceptual learning while maintaining functional communication.
**Multimodal Integration Control**: Machine learning models can optimize the balance between auditory enhancement and supplementary visual or tactile information based on individual multimodal integration patterns. By understanding how each user integrates information across sensory modalities, these systems can provide optimally balanced multisensory support.

### 5.3. Integrated Bidirectional Communication Systems

The ultimate goal of this bidirectional approach is to develop comprehensive communication neuroprosthetic systems that address both speech production and auditory processing within a unified framework. Such systems would provide closed-loop support across the entire communication cycle.

#### 5.3.1. Synchronized Enhancement of Production and Perception

Integrated systems can synchronize the enhancement of speech production and auditory processing to maximize communicative effectiveness. For example, by detecting the neural signatures of speech planning, the system could preemptively enhance auditory processing to optimize self-monitoring during speech production.

AI enables this synchronized enhancement through:

**Cross-Domain State Prediction**: Deep learning models can predict upcoming states in one domain (e.g., speech production) based on current states in another domain (e.g., auditory attention), allowing preemptive optimization across domains.
**Joint Parameter Optimization**: Reinforcement learning algorithms can optimize stimulation parameters across production and perception domains simultaneously, potentially identifying synergistic parameter combinations that exceed the efficacy of independently optimized approaches.
**Conversation State Modeling**: Natural language processing models can track conversation state (e.g., speaking vs. listening, question vs. response) to dynamically reconfigure the system for the current communicative context. This context awareness enables more intelligent switching between production-focused and perception-focused enhancement.

#### 5.3.2. Personalized Communication Training

Beyond immediate enhancement, integrated systems can provide personalized training to improve natural communication abilities over time.

AI enables sophisticated communication training through:

**Individualized Learning Trajectories**: Machine learning algorithms can model individual learning patterns and design optimal training progressions that maximize transfer to natural communication contexts.
**Adaptive Scaffolding**: Deep reinforcement learning approaches can implement optimal scaffolding strategies, gradually reducing enhancement as natural abilities improve. This approach ensures that users develop maximal independent communication skills while maintaining functional communication throughout the process.
**Social Context Adaptation**: Natural language processing and computer vision models can detect social context cues and adjust enhancement parameters to optimize communication in different social settings. This context sensitivity recognizes that communication demands vary across different social environments and relationships.

#### 5.3.3. Implementation Considerations Technical and Ethical Implementation Framework

The successful implementation of integrated bidirectional systems requires careful consideration of several factors:

**Hardware Integration**: Developing hardware that can simultaneously record neural signals and deliver stimulation without interference presents significant engineering challenges. Recent advances in artifact rejection algorithms and hardware design are beginning to address these challenges, but further innovation is needed.
**Computational Efficiency**: The real-time operation of sophisticated AI models on portable hardware requires substantial optimization. Techniques such as model quantization, pruning, and hardware acceleration will be essential for practical implementation.
**User Interface Design**: Creating intuitive interfaces that give users appropriate control while minimizing cognitive load is crucial for acceptance and effective use. Participatory design approaches involving end-users throughout the development process will be essential for creating truly usable systems.
**Ethical Considerations**: Ensuring user autonomy, data privacy, and appropriate risk management requires careful attention to ethical dimensions throughout the development process. The intimate nature of these technologies, which interact directly with neural processes underlying communication, raises important questions about agency, identity, and privacy that must be thoughtfully addressed.

### 5.4. Case Examples: Potential Applications Across Communication Disorders

To illustrate the potential of bidirectional AI-guided NIBS for communication disorders, consider the following case examples:

#### 5.4.1. Post-Stroke Aphasia

For individuals with post-stroke aphasia, an integrated system would combine several innovative approaches working in concert. Transcranial direct current stimulation would target left perilesional language areas, enhancing the neural signal quality crucial for effective speech decoding while simultaneously promoting neuroplasticity in damaged regions. This stimulation works alongside AI-optimized decoding systems that interpret attempted speech from both electroencephalography and residual muscle activity, providing an immediate voice for those struggling to communicate naturally.

The system’s sophistication emerges in its closed-loop functionality, continuously adjusting stimulation parameters based on neural markers that indicate speech planning difficulties. When the brain struggles to formulate language, the system responds with calibrated support. Simultaneously, adaptive auditory processing enhancement optimizes the individual’s comprehension of conversational partners, addressing both expressive and receptive communication. Throughout rehabilitation, personalized training protocols gradually increase communication complexity while maintaining functional success, ensuring the patient experiences meaningful progress without frustration.

This comprehensive approach offers immediate communication support while simultaneously promoting the recovery of natural speech abilities through targeted neuroplasticity induction, addressing both short-term needs and long-term rehabilitation goals.

#### 5.4.2. Age-Related Hearing Loss with Mild Cognitive Impairment

For older adults navigating the challenging intersection of hearing loss and cognitive impairment, an integrated system addresses multiple dimensions of communication breakdown. Transcranial alternating current stimulation targets both auditory and prefrontal regions, enhancing fundamental auditory processing while strengthening the cognitive control necessary for complex communication. This neurophysiological support works in tandem with AI-driven speech enhancement algorithms specifically optimized for individual hearing profiles, acknowledging the unique pattern of auditory strengths and deficits each person presents.

The system continuously adapts by monitoring neural markers of comprehension and cognitive load, adjusting stimulation parameters when signs of communication strain emerge. Visual reinforcement through an auxiliary display highlights key information in decoded speech, providing multimodal support when auditory processing falters. Beyond immediate communication support, the system integrates adaptive cognitive training with communication practice, strengthening underlying cognitive abilities essential for successful interaction.

This multifaceted approach addresses both the sensory and cognitive components of communication difficulty in this population, potentially maintaining vital social engagement and cognitive stimulation that might otherwise diminish as communication becomes increasingly challenging.

#### 5.4.3. Developmental Language Disorder

For children with developmental language disorders, a developmentally appropriate system creates a supportive environment for language acquisition during critical formative years. Low-intensity transcranial direct current stimulation targets language regions during structured activities, carefully calibrated for developing neural systems. Children engage through gamified interfaces that transform therapeutic exercises into motivating play experiences, maintaining the engagement essential for effective neuroplasticity.

Artificial intelligence continuously monitors engagement and learning states, optimizing stimulation timing to coincide with moments of focused attention and receptivity. The system adapts difficulty progression based on a sophisticated analysis of both performance metrics and neural markers indicating learning consolidation. Parents and clinicians receive guidance through specialized interfaces that suggest supportive communication strategies aligned with the child’s current developmental trajectory and therapeutic focus.

This application exemplifies the potential for AI-guided NIBS to enhance traditional therapeutic approaches by creating optimal conditions for neuroplasticity during critical developmental periods, potentially altering developmental trajectories through precisely timed neural intervention combined with engaging, meaningful communication experiences.

### 5.5. Future Directions and Challenges

While the bidirectional application of AI-guided NIBS shows tremendous promise for communication disorders, several challenges must be addressed to realize this potential:

**Long-term Safety and Efficacy**: Establishing the safety and efficacy of chronic or repeated NIBS requires longitudinal studies. Current evidence primarily addresses short-term effects, leaving questions about cumulative impacts and potential adaptive changes with prolonged use.
**Practical Usability**: Translating laboratory demonstrations to practical, user-friendly systems requires significant attention to form factor, reliability, and ease of use. The complex capabilities described must ultimately be packaged in systems that can be operated independently by users or caregivers.
**Individual Variability**: The substantial inter-individual variability in response to NIBS presents both a challenge and an opportunity. While this variability complicates standardization, it also highlights the potential value of AI-driven personalization.
**Regulatory Pathways**: Establishing appropriate regulatory frameworks for adaptive AI-guided neuromodulation systems presents novel challenges. The self-modifying nature of adaptive AI systems does not fit neatly into traditional medical device regulatory paradigms, necessitating innovative approaches to ensuring safety while enabling beneficial innovation.

The integration of NIBS and AI for communication disorders represents a highly promising frontier, with the potential to address the limitations of current approaches through personalization, adaptation, and bidirectional support across the communication cycle. The bidirectional framework presented here provides a roadmap for future research and development efforts aimed at creating more effective, natural, and personalized communication neuroprosthetic systems.

## 6. Implementation Considerations

The successful implementation of bidirectional communication neuroprosthetics using AI-guided non-invasive brain stimulation requires addressing several critical technical and biological challenges. This section highlights the most essential considerations for translating the proposed framework into practical applications.

### 6.1. Technical Considerations

#### 6.1.1. Precision Targeting in Communication Networks

Effective implementation requires the accurate targeting of speech and auditory processing networks. Computational models derived from individual neuroimaging can predict current flow patterns through heterogeneous head tissues, significantly improving targeting precision. Huang et al. [47] demonstrated that individualized head models can account for anatomical variations that affect current distributions during stimulation. For communication applications, this personalized approach is particularly important due to the significant inter-individual variability in language network organization.

Advanced electrode configurations can further enhance spatial specificity. High-definition tDCS using small electrodes in specific montages has shown superior spatial focality compared to conventional approaches. For auditory applications, Richardson et al. [52] demonstrated improved temporal lobe targeting through specialized electrode configurations that account for the orientation of the auditory cortex within the Sylvian fissure.

#### 6.1.2. System Integration Challenges

A critical implementation challenge is minimizing interference between stimulation and neural recording components. Time-division multiplexing, where stimulation and recording alternate in rapid succession, offers one solution. Kohli and Casson [53] implemented a system that switches between stimulation and recording at millisecond time scales, minimizing interference while maintaining effective operation.

Advanced signal processing techniques can complement hardware solutions. Adaptive filtering approaches show particular promise for real-time applications. Yang et al. [54] demonstrated a real-time artifact removal system using adaptive filters that can dynamically respond to changing artifact characteristics during stimulation.

#### 6.1.3. Usability and Form Factor

For practical adoption, systems must be compact, comfortable, and intuitive. Recent advances in dry electrode technology can maintain acceptable signal quality while eliminating conductive gel, significantly improving usability. Behind-the-ear EEG systems and miniaturized stimulation devices weighing under 100 g enable truly portable applications suitable for daily use [55,56].

Software architecture must support the real-time processing and seamless integration of multiple components. Edge computing approaches can reduce latency by distributing processing across system components. Raut and Andreou [57] demonstrated a distributed BCI system that performed initial signal processing on headset microcontrollers before sending extracted features to a smartphone, achieving a 60% reduction in latency compared to raw data transmission.

### 6.2. Biological Considerations

#### 6.2.1. Neuroplasticity Mechanisms and Timing

Targeting the appropriate neuroplastic mechanisms is essential for optimizing intervention efficacy. Hebbian plasticity, characterized by the strengthening of synaptic connections between neurons that fire together, plays a crucial role in speech motor learning. Hickok et al. [58] showed that the repeated co-activation of articulatory motor neurons and corresponding auditory feedback circuits strengthens the sensorimotor integration pathways critical for speech production.

The timing of interventions significantly impacts efficacy through time-dependent plasticity mechanisms. Saur et al. [59] identified distinct phases of plasticity following stroke-induced aphasia, with a subacute reorganization phase demonstrating heightened responsiveness to intervention. For developmental communication disorders, Ciaccio et al. [60] found that interventions delivered during natural developmental transitions in speech motor control (ages 4–6 years) produced larger and more lasting improvements.

#### 6.2.2. Individual Variability and Response Prediction

The substantial inter-individual variability in response to brain stimulation necessitates personalized approaches. Genetic variations affecting neuroplasticity pathways contribute significantly to this variability. Plewnia et al. [48] demonstrated that carriers of the BDNF Val66Met polymorphism showed attenuated responses to anodal tDCS over language areas compared to non-carriers.

Several biomarkers show promise for predicting individual response to neuromodulation. Structural connectivity patterns provide one class of predictive markers; Basal et al. [61] found that fractional anisotropy values in left language pathways significantly predicted response to tDCS in chronic aphasia patients. Functional connectivity between the left inferior frontal and posterior temporal regions has similarly demonstrated predictive value for treatment outcomes [62].

#### 6.2.3. Etiology-Specific Adaptations

Different etiologies of communication disorders require tailored implementation approaches. In post-stroke aphasia, the presence of focal lesions alters current flow patterns during stimulation. Wagner et al. [63] demonstrated that lesioned tissue can create irregular current distributions that may diminish stimulation efficacy at intended targets. These findings highlight the importance of lesion-aware targeting approaches.

For neurodegenerative conditions like Primary Progressive Aphasia, Nissim et al. [64] found that patients showed initial positive responses to stimulation but required more frequent “booster” sessions to maintain improvements compared to stroke patients. This accelerated decay of benefits likely reflects ongoing neurodegeneration counteracting stimulation-induced plasticity.

Developmental disorders operate within distinct neurobiological contexts. Wilkinson et al. [65] observed that children with developmental speech disorders showed enhanced responsiveness to tDCS compared to adults with acquired disorders, achieving comparable improvements with lower stimulation intensities. This heightened responsiveness likely reflects the greater baseline plasticity of developing neural systems but also necessitates more conservative approaches regarding safety.

### 6.3. Implementation Roadmap

Translating the bidirectional framework from concept to clinical application requires a phased approach:

1. **Initial Development Phase**: Create proof-of-concept systems integrating neural recording, AI-driven signal processing, and NIBS in laboratory settings. Validate component interactions and optimize parameters using healthy participants.
2. **Clinical Validation Phase**: Test optimized systems with specific communication disorder populations, starting with structured communication tasks in controlled environments and progressing to more naturalistic scenarios. Implement multimodal biomarker identification to develop response prediction models.
3. **Adaptation and Refinement Phase**: Refine systems based on user feedback and clinical outcomes, with a particular focus on usability improvements and the development of adaptive algorithms that learn from individual usage patterns. Explore telehealth integration for remote monitoring and adjustment.
4. **Translation to Practice**: Develop clinical protocols, training programs, and technical standards to support broader clinical implementation. Collaborate with regulatory bodies to establish appropriate pathways for approval and coverage.

Throughout this process, the bidirectional nature of the system should be preserved, with equal attention to both speech production and auditory processing enhancements. The integration of these components under unified AI control represents the core innovation of this approach and the key to addressing the complete communication cycle for individuals with diverse communication disorders.

## 7. Conclusion: Future Directions and Potential Impact

The bidirectional approach to communication neuroprosthetics presented in this perspective represents a significant conceptual advance in addressing the complex challenges faced by individuals with speech and hearing impairments. By integrating non-invasive brain stimulation with artificial intelligence and targeting both speech production and auditory processing domains, this framework offers a more comprehensive solution than traditional unidirectional approaches.

### 7.1. Future Research Directions

Several key research priorities emerge from this bidirectional framework:

**Integrated System Development and Validation**: The most immediate research priority is the development and validation of fully integrated systems that seamlessly combine neural recording, stimulation, and AI components for both speech and auditory processing. Initial studies should focus on demonstrating the feasibility and efficacy of switching between speech production enhancement and auditory processing enhancement in response to communication context. Comparative studies examining the bidirectional approach versus single-domain interventions will be essential for quantifying the added value of integration.
**Optimizing Cross-Domain Interactions**: Further research should explore how interventions in one communication domain might impact the other. The potential synergistic effects between speech production and auditory processing enhancement deserve particular attention. For example, studies might investigate whether enhancing auditory feedback processing through tACS can indirectly improve speech motor learning during subsequent tDCS-enhanced articulation training. Understanding these cross-domain interactions could lead to more efficient intervention protocols that leverage natural connections between production and perception.
**Longitudinal Studies of Neuroplastic Effects**: The long-term neuroplastic effects of bidirectional interventions require careful investigation. Studies examining whether alternating between speech and auditory enhancement produces different long-term network reorganization compared to domain-specific interventions could reveal important mechanisms for maximizing therapeutic benefits. The potential for bidirectional approaches to induce more naturalistic and functional neural reorganization through supporting complete communication cycles represents a particularly promising direction.
**Expanded Application to Diverse Populations**: Future research should explore the applicability of the bidirectional framework across diverse communication disorders, including developmental conditions, neurodegenerative diseases, and trauma-induced impairments. Specific adaptations for pediatric populations, who may benefit particularly from early bidirectional support due to critical periods in communication development, represent an especially promising direction. Studies examining how the optimal balance between speech and auditory enhancement might differ across various disorders and developmental stages will be essential for maximizing clinical utility.
**Technological Miniaturization and Integration**: Advancing the technological implementation toward more compact, user-friendly systems represents a critical engineering challenge. Research on flexible electronics, energy-efficient computing, and seamless user interfaces will be essential for translating the bidirectional framework into practical, everyday tools. Particular attention should be directed toward developing systems that can operate reliably in natural environments rather than controlled laboratory settings.

### 7.2. Potential Impact

The potential impact of bidirectional communication neuroprosthetics extends across multiple domains:

**Clinical Impact**: For individuals with communication disorders, the bidirectional approach offers the possibility of more natural, effective, and comprehensive support than current unidirectional methods. By addressing both expressive and receptive communication simultaneously, these systems could potentially reduce the cognitive load associated with communication, allowing users to focus more on content and social interaction rather than the mechanics of production or comprehension. The personalized nature of AI-guided approaches could further enhance outcomes by optimizing interventions for individual neuroanatomy, functional organization, and symptom profiles.
**Scientific Understanding**: Beyond direct clinical applications, the development of bidirectional systems will likely advance our scientific understanding of the neural bases of communication. The closed-loop interaction between production and perception systems, particularly when modulated by non-invasive stimulation, provides a unique window into natural communication processes. Research in this area may yield insights into the dynamic interplay between speaking and listening networks that could inform basic neuroscience research on language and communication.
**Accessibility and Inclusion**: By focusing on non-invasive approaches, the bidirectional framework has the potential to substantially expand access to communication neuroprosthetics. Unlike invasive alternatives that require neurosurgery, these systems could potentially be implemented in diverse clinical settings and potentially even home environments with appropriate training and supervision. This accessibility could help address disparities in intervention access, particularly for underserved populations and regions with limited specialized medical infrastructure.
**Quality of Life Enhancement**: The ultimate impact of bidirectional communication support extends beyond functional communication to broader quality of life. Communication is fundamental to social connection, educational opportunities, vocational success, and psychological wellbeing. By supporting the complete communication cycle in a personalized, adaptive manner, bidirectional systems could help restore these connections for individuals with communication disorders, potentially reducing isolation and enhancing participation across life domains.

### 7.3. Concluding Remarks

The integration of non-invasive brain stimulation with artificial intelligence to create bidirectional communication neuroprosthetics represents a promising frontier in rehabilitation technology. While significant technical and biological challenges remain, the conceptual framework presented here offers a roadmap for addressing these challenges through interdisciplinary collaboration among neuroscientists, engineers, clinicians, and individuals with communication disorders.

The bidirectional nature of human communication—the continuous interplay between expression and reception—has long been recognized in theoretical models of language but has not been fully reflected in technological interventions. By aligning our assistive approaches more closely with the natural structure of communication, we may develop more effective, intuitive, and beneficial tools for those whose communication abilities have been affected by injury, disease, or developmental conditions.

As this field advances, maintaining a balance between technological innovation and human-centered design will be essential. The ultimate measure of success for bidirectional communication neuroprosthetics will not be technical sophistication but rather the degree to which they enable individuals to engage in meaningful, natural communication and participate fully in their communities. With continued research and development guided by this principle, bidirectional approaches have the potential to transform the landscape of communication intervention and significantly enhance the quality of life for those with communication disorders.
